# Second hand tobacco smoke adversely affects the bone of immature rats

**DOI:** 10.6061/clinics/2017(12)11

**Published:** 2017-12

**Authors:** Rodrigo César Rosa, Sângela Cunha Pereira, Fabrizio Antônio Gomide Cardoso, Abadio Gonçalves Caetano, Hildemberg Agostinho Rocha de Santiago, José Batista Volpon

**Affiliations:** I Departamento de Biologia Estrutural, Universidade Federal do Triangulo Mineiro, Uberaba, MG, BR; II Graduacao, Faculdade de Medicina, Universidade de Uberaba, Uberaba, MG, BR; III Departamento de Biomecanica, Medicina e Reabilitacao do Aparelho Locomotor, Faculdade de Medicina de Ribeirao Preto, Universidade de Sao Paulo, Ribeirao Preto, SP, BR

**Keywords:** Smoking, Bone Development, Bone Density, Tibia, Rats

## Abstract

**OBJECTIVES:**

To evaluate the influence of secondhand cigarette smoke exposure on longitudinal growth of the tibia of growing rats and some parameters of bone quality.

**METHODS:**

Forty female rats were randomly divided into four groups: control: rats were sham exposed; 30 days: rats were exposed to tobacco smoke for 30 days; 45 days: rats were exposed to tobacco smoke for 45 days; and 60 days: rats were exposed to tobacco smoke for 60 days. Blood samples were collected to evaluate the levels of cotinine and alkaline phosphatase. Both tibias were dissected and weighed; the lengths were measured, and the bones were then stored in a freezer for analysis of bone mineral content and mechanical resistance (maximal load and stiffness).

**RESULTS:**

Exposure of rats to tobacco smoke significantly compromised bone health, suggesting that the harmful effects may be time dependent. Harmful effects on bone growth were detected and were more pronounced at 60-day follow-ups with a 41.8% reduction in alkaline phosphatase levels (*p*<0.01) and a decrease of 11.25% in tibia length (*p*<0.001). Furthermore, a 41.5% decrease in bone mineral density was observed (*p*<0.001), leading to a 42.8% reduction in maximum strength (*p*<0.001) and a 56.7% reduction in stiffness (*p*<0.001).

**CONCLUSION:**

Second hand cigarette smoke exposure in rats affected bones that were weaker, deforming them and making them osteopenic. Additionally, the long bone was shorter, suggesting interference with growth. Such events seem to be related to time of exposure.

## INTRODUCTION

Chronic tobacco smoke inhalation has an important impact on different organs and systems and is strongly associated with an increased incidence of certain types of cancer 1. Tobacco smoke can reach the respiratory system by active inhalation or passively when non-smoking people are exposed to a polluted environment (second hand smoke). In both types of exposure, the smoke travels into the lungs, and then, via the blood circulation, the smoke reaches different organs, disturbing metabolic routes and compromising the individual’s health 2.

Tobacco cigarette smoke consists of two components: central or mainstream smoke and peripheral or sidestream smoke. The first component results from burning the tobacco at high temperatures (above 950° C) and the passage of smoke through the tobacco column and filter followed by active inhalation by the smokers 3. The second component, sidestream cigarette smoke, is generated at lower temperatures (approximately 350° C) during the slow and spontaneous smoldering of a cigarette, cigar, pipe or fire in a closed environment. Importantly, this type of smoke contains four times more harmful compounds than the main strain smoke, 4 and non-smokers may be exposed to an environment contaminated by so-called second hand smoke (result of expelled smoke and smoke from smoldering burning).

It has been demonstrated both clinically and experimentally that tobacco smoke alters bone quality 5 and bone healing 6-8, and in some cases, it is associated with a disturbance in fracture healing 9-11. Additionally, there are some reports that suggest that nicotine causes a decrease in weight and body mass index 12. Children, during the growth period, undergo important physiological events that may be hazardously affected by tobacco smoke. Therefore, the present study aims to evaluate the influence of second hand cigarette smoke exposure on longitudinal growth of the tibia in growing rats and on some bone quality parameters.

## METHODS

The experimental protocol was carried out according to the Guide for the Care and Use of Laboratory Animals (National Research Council - USA, 2011) and approved by the Ethics Committee on Animal Experimentation of the School of Medicine of Ribeirão Preto, the University of São Paulo, Brazil (Protocol n. 139/2013).

Forty female rats (*Rattus norvegicus albinus var. Wistar*) were obtained from the vivarium of the university and housed under standard laboratory conditions (room temperature 22±2° C, humidity 55±5%, 12 h light-dark cycles) with free access to tap water and chow (Nuvilab CR-1, Colombo, PR, Brazil). The rats weighed 300 to 350 g (80–100 days old), were kept in the laboratory environment for one week for acclimatization and then were randomly distributed into four groups: control: rats were sham exposed; 30 days: rats were exposed to tobacco smoke for 30 days; 45 days: rats were exposed to tobacco smoke for 45 days; and 60 days: rats were exposed to tobacco smoke for 60 days. All animals were housed in standard laboratory cages, with the same number of animals per box, allowing similar gait activities. The animals were inspected daily and weighed weekly. Euthanasia was performed with an overdose of thiopental sodium, injected intraperitoneally. Blood samples were immediately collected by cardiac puncture to evaluate the levels of cotinine and alkaline phosphatase. Both tibias were dissected and weighed; the lengths were measured, and the bones were stored in a freezer for future analysis (bone mineral content and mechanical resistance).

### Exposure to Tobacco Smoke

To expose the rats to cigarette smoke, a special device was constructed based on previous publications 13-15. Details of the equipment that we used can be found in the paper by Santiago et al. 11. In summary, there were four cylindrical compartments to accommodate one rat in each compartment. One end of the cylinder was open and used to manipulate the animal. The other end was funnel-shaped and used to fit the animal muzzle and to introduce and clear the smoke. From a burning cigarette, a peristaltic pump (Provitec AWG 5,000 AX-D, São Paulo, SP, Brazil) aspirated the smoke and transferred it to a distribution chamber and then to the animals’ compartments. A timer controlled the introduction of smoke for 15 s and its substitution with clean air for 30 s, thus completing one cycle. After the exposure, the smoke was exhausted to the outside. The first seven days were used to adapt the rat to smoke exposure and consisted of burning a cigarette twice a day (morning and afternoon), with a six-hour interval between exposures. The following week, the rats were exposed to the smoke of two cigarettes in the morning and two cigarettes in the afternoon, with a six-hour interval. The period of exposure to tobacco was established according to the experimental groups: 30 days, 45 days and 60 days.

Every seven days, the percentage of carbon monoxide within each cylindrical chamber was measured with a portable carbon monoxide meter (Instrupemp, ITMCO-1500, São Paulo, SP, Brazil). The four chambers presented similar levels of carbon monoxide (338.79±1.16 ppm). The source of the smoke was Marlboro cigarettes (Phillips Morris, Santa Cruz do Sul, RS, Brazil), with each unit containing the following according to the product label: 0.8 mg of nicotine, 10 mg of tar and 10 mg of carbon monoxide.

### Analysis of alkaline phosphatase activity in blood plasma

Following euthanasia, blood samples were collected in heparinized tubes that were centrifuged at 1831 g for 10 minutes. Alkaline phosphatase levels were obtained in the plasma fraction using a commercial kit (Roche, Jacarepaguá, RJ, Brazil). The absorbance was read at 410 nm in a spectrophotometer (COBAS Integra, Jacarepaguá, RJ, Brazil). The results were expressed in UI, corresponding to 0.01667 µkat/L.

### Level of cotinine in the serum

Cotinine in the serum was obtained by heart puncture and processed according to previously described methods 16-19, using a method with a specificity of 97.4% and a sensitivity of 96.3%. A cotinine serum concentration ranging from 2.1 to 17.5 ng/mL was accepted as the cut-off level to characterize the animal exposure as secondhand smoke, similar to levels found in human passive smokers 20,21. Cotinine is a metabolic product of nicotine, with a low rate of metabolism and renal excretion, so that its half-life is ten times longer than that of nicotine. In addition, cotinine remains constant if smoking persists 22, so it is a useful marker for assessing tobacco addiction.

### Bone length

The lengths of the tibias were obtained with a digital caliper by three consecutive measurements and an average calculation.

### Bone mineral density

Bone mineral density was determined by dual-energy X-ray absorptiometry (DXA) using a Lunar DPX-IQ densitometer (Lunar; software version 4.7e, GE Healthcare, Chalfont St. Giles, United Kingdom) with software for small samples. The tibias were immersed in ethanolin a small container, and scanning of the entire bone was carried out. Then, the region of interest was delimited by a square measuring 0.90 cm^2^, taking the tibial tuberosity as an anatomical landmark.

### Mechanical Testing

The entire bone was tested in a 3-point flexion. The bone extremities were rested on two metallic supports that were 25 mm apart, and a progressive load was vertically applied at the center of the posterior surface of the bone at a constant displacement rate of 1 mm/min until failure. The testing device (EMIC, São José dos Pinhais, PR, Brazil) was equipped with a 500 N load cell, and the load-deflection curve was obtained in real time. The maximal load and stiffness were calculated using software (TESC software, version 13.4, São José dos Pinhais, PR, Brazil).

### Statistical Analysis

The SPSS program (SPSS for Windows - Version 11.0 - SPSS Inc.) was used for statistical analysis. Data were initially submitted to descriptive analysis, with a calculation of means and standard deviations. After checking for normality (ANOVA), the level of significance was determined by Tukey’s test, with a significance level of 5% (*p*<0.05).

## RESULTS

**Figure 1:**
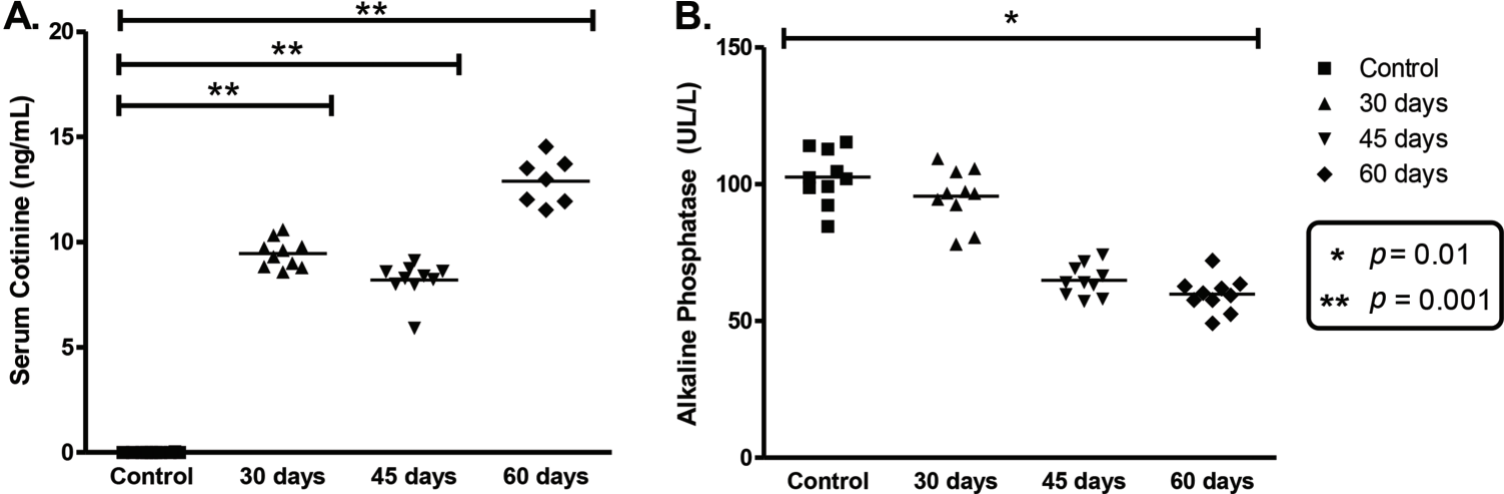
Biochemical data. A. Levels of cotinine in blood plasm; B. Average of alkaline phosphatase activity in blood plasma. The asterisks indicate comparisons with significant differences.

The levels of cotinine in the blood plasma were significantly lower (*p*<0.001) in the control group (0.0006±0.008 ng/mL) compared to the groups with30 (9.5±0.8 ng/mL), 45 (8.2±0.85 ng/mL) and 60 days (13.2±1.2 ng/mL) of exposure to tobacco smoke (Figure 1).

The alkaline phosphatase activity in blood plasma after 60days of exposure was reduced by 41.8% in relation to the control group (*p*<0.01), but with no significant difference among the exposed groups (*p*>0.05) (Figure 1).

**Figure 2:**
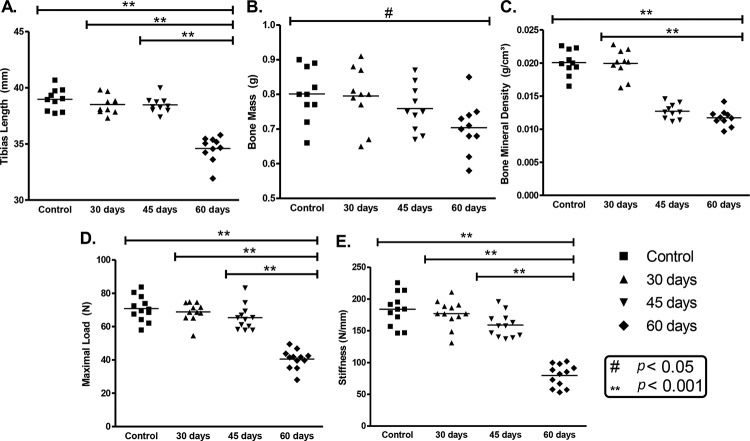
Analysis of skeletal development, bone mineral content and mechanical resistance. A. Tibia lengths; B. bone mass; C. bone mineral density; D. maximal load; E. stiffness. The asterisks indicate comparisons with significant differences.

The mean tibia length from animals with 60days of exposure was 11.25% shorter than in the control group (*p*<0.001). Additionally, the comparison of the 30 days (1.21%) and 45 days (1.28%) of exposure groups with the 60days of exposure group showed significant differences (*p*<0.001) (Figure 2).

The mass of the tibias showed a significant difference between the control and the 60-day group (*p*<0.05), but not among the exposed groups (*p*>0.05) (Figure 2).

The mean bone mineral density of the tibias after 60 days of exposure was statistically reduced by 41.5% in comparison with the control animal group. There was also a significant difference between the 30-day exposure and the 45- and 60-day exposure groups (*p*<0.05), but no difference between the 45- and 60-day exposure groups (*p*>0.05) (Figure 2).

The mean maximal load of the tibias from animals exposed to tobacco smoke for 60 days was statistically reduced by 42.8% in comparison with the control animals (*p*<0.001). There was also a significant difference between the 30-day and 60-day exposure groups as well as between the 45-day and 60-day exposure groups (*p*<0.05) (Figure 2).

The mean stiffness of the tibias from the 60-day exposure groups was significantly decreased by 56.7% in comparison with the control animals (*p*<0.001). There was also a significant difference between the 30-day and 60-day exposure groups, as well as between the 45-day and 60-day exposure groups (*p*<0.05) (Figure 2).

## DISCUSSION

It has been fully demonstrated that addiction to tobacco products compromises general health because it affects the function of many organs and systems and is closely associated with the incidence of cancer and cardiovascular diseases. In the skeletal system, bone quality and fracture healing are compromised 11,23-25. However, even non-smokers may be affected by chronic exposure to indoor smoke expelled by active smokers (mainstream smoke) and by smoldering cigarettes (sidestream smoke). The second hand smokers correspond to those individuals who do not smoke but breathe air polluted with tobacco smoke. In addition, the environmental tobacco smoke may impregnate surfaces of furniture, floor, animal fur, towels and bed linens. The toxicity of cigarette smoke increases with aging and length of exposure 26. Moreover, children undergo important physiological changes that are unique during the growth period and that may be hazardously affected by the tobacco smoke.

Our results showed that exposure of rats to secondhand tobacco smoke significantly compromised bone health, as demonstrated by the negative repercussions in all of the studied parameters. Further, our results suggest that the harmful effects may be time dependent. However, because the follow up time in our research was 60 days, it was not possible to establish the long-term effects of such exposure. Further, we used immature animals, making it possible to detect the detrimental effects on bone growth. These effects were more pronounced at 60-day follow-ups with a reduction of 11.25% in tibia lengths and may be related to the fact that nicotine interferes with the formation of signaling molecules, leading to changes in metabolism and bone growth 27. However, tobacco smoke contains many other potentially hazardous substances that may affect bone metabolism, thus reinforcing the deleterious effects of nicotine on bone.

As expected, the aforementioned characteristics are confirmed by the results of mechanical tests. Thus, tibias from exposed animals were weaker and less rigid. Stiffness is a parameter that represents how the bone deforms under load, and the maximal load refers to bone resistance to deformation. These results are consistent with a study that demonstrated a reduction in the mechanical resistance of bones in rats that underwent subcutaneous injections of nicotine 28.

Our current results show a reduction of 41.8% in bone mineral density and 56.7% in stiffness, due to metabolic changes caused by tobacco exposure. The metabolic changes were expressed by a reduction of 41.8% in plasma concentrations of alkaline phosphatase for the 60-day group. Because the majority of serum alkaline phosphatase in the growing period is of skeletal origin 29, these findings may also reflect a depression in bone metabolism. Additionally, previous studies showed the effects of cigarette smoke on cellular and gene expression. Such studies point to a delay in the chondrogenesis of fracture callus 30, osteoblastic gene expression 31 and modulation of osteoprogenitor cells 32.

Finally, there are some limitations in our experimental design that may warn against a strict translation of our conclusions to human cases. Our model of second-hand exposure has not been confirmed to mimic what occurs in humans. We chose the interval ranging from 2.1 to 17.5 ng/mL as the cut-off range of cotinine levels to characterize animal exposure to second hand smoke. However, these data were validated in human second-hand smokers, and there is no available information for the rat. Additionally, we were unable to find an ideal environment in which to expose the animals. Two main models are available. In one, the animals are exposed in a collective chamber that is successively filled and exhausted with tobacco smoke 13,14. In this model, the rat fur is impregnated with smoke substances that are ingested when the animals self-clean. Eye irritation also occurs in this model. The second device is based on individual chambers in which the muzzle is exposed, and the effects of fur impregnation do not occur. However, both models present drawbacks because they are not true models of secondhand smoke exposure. Lastly, the smoke in the cameras is characterized as mainstream smoke, which is different than the mixed sidestream and mainstream smoke that is usually found indoors and inhaled by second hand smokers.

In summary, our results suggest that exposure of rats to secondhand smoke adversely affects bone structure. Such results are in accordance with those of Santiago et al. 11. Most importantly, smoke also affects bone growth. To the best of our knowledge, this finding was not specifically reported before, but it calls for further basic investigation and clinical studies. However, our results are strong enough to alert health authorities to pass and enforce laws against smoking in indoor environments.

Second hand cigarette smoke exposure to rats affected bones that were weaker, deforming them and making them osteopenic. Additionally, the long bone was shorter, suggesting interference with growth. Such events seem to be related to the duration of exposure.

## AUTHOR CONTRIBUTIONS

Rosa RC was responsible for the design, and intellectual and scientific content of the study, technical procedures, acquisition and interpretation of data, manuscript writing, critical revision, and approval of the final version of the manuscript. Pereira SC was responsible for the technical procedures, acquisition and interpretation of data. Cardoso FA was responsible for the acquisition and interpretation of data, statistical analysis, and manuscript revision. Caetano AG was responsible for the acquisition and interpretation of data, and technical procedures. de Santiago HA was responsible for the technical procedures and acquisition of data. Volpon JB was responsible for the design, and intellectual and scientific content of the study, manuscript writing, critical revision and final approval of the manuscript, and securing funding.
